# Ethically Researching Health and Disaster: Lessons from over a Decade of Research since the 3.11 Disaster in Fukushima

**DOI:** 10.31662/jmaj.2023-0145

**Published:** 2024-02-27

**Authors:** Kaori Honda, Sudeepa Abeysinghe, Claire Leppold, Alison Lloyd Williams, Akihiko Ozaki, Aya Goto

**Affiliations:** 1 Center for Integrated Science and Humanities, Fukushima Medical University, Fukushima, Japan; 2 Global Health Policy Unit, University of Edinburgh, Edinburgh, UK; 3 Child & Community Wellbeing Unit, Centre for Health Equity, Melbourne School of Population and Global Health, University of Melbourne, Carlton, VIC, Australia; 4 Sociology Department, Bowland College, Lancaster University, Lancaster, UK; 5 Department of Breast and Thyroid Surgery, Jyoban Hospital of Tokiwa Foundation, Iwaki, Japan; 6 Department of Thyroid and Endocrinology, Fukushima Medical University, Fukushima, Japan

**Keywords:** ethics, 3.11, Fukushima, disaster

Health research around disaster is an expanding field, reflecting the frequency and severity of climate-related events as well as other forms of disaster. After a disaster occurs, affected populations and places become prominent sites of research activity. Such activities have the potential to benefit communities but can also be a cause of further stress. There continues to be a lack of consensus around the practical ethics of health and disaster research ^(1)^, prompting further reflection on what constitutes contextually-sensitive ethical practice in this field.

It has been over 10 years since the Fukushima 3.11 “triple disaster” of earthquake, tsunami, and nuclear disaster. While the health, social, and political impacts of 3.11 continue ^(2)^, the time that has elapsed since the acute disaster phase has allowed for broader reflection around the research strategies that occurred in order to adapt these for future events. Following a series of interviews with researchers in July 2023, the project “Ethically Researching Health and Disasters: 3.11 and Beyond” brought together researchers at Fukushima Medical University to discuss learnings from the past decade of 3.11 health research. It is essential to assess the impacts of research activities following 3.11 ^(3)^ to bring about practical recommendations for future health and disaster research. Here, we report the main discussion points from the meeting (please see also Table 1).

**Table 1.** Lessons for Ethical Research Practice from the 3.11 Disaster in Fukushima.

Issues	Recommendations
Communities	・Timely and meaningful feedback to research participants
・Research fatigue in the local communities	・Capacity building of researchers in communicating with communities and a more participatory approach
Researchers	・Facilitate researchers’ understanding of the risk of vicarious trauma
・Vicarious trauma among researchers	・Facilitate the public’s understanding of the meaning of research
・The disaster affected local researchers	
Bridging issue	・Recognize the resilience of people and communities in disaster recovery
・Stigmatizing by positioning the disaster-affected population as vulnerable	

A key discussion point surrounded the experiences of the local communities. Research fatigue, especially survey fatigue, has been pronounced across populations in the Fukushima Prefecture. Continuous probing on mental wellbeing in particular―which to an extent have continued over time following the disaster―exacerbate stress for a population already experiencing a high degree of uncertainty. Many researchers also raised the importance of timely and meaningful feedback to research participants in these contexts. Feedback can be considered an ethical necessity ^(4)^, helps to maintain transparent and trusting relationships between communities and researchers, and further facilitates the application of research findings to health system and service improvements. In such a context, the capacity building of researchers in communicating with communities and a more participatory approach are critical ^(5)^.

Support for researchers also requires further consideration. The problem of vicarious trauma―the potential of researchers to experience distress as a result of prolonged exposure to the traumatic stories of participants and members of the disaster-impacted community―is becoming more widely discussed in relation to disaster research ^(6)^. Creating structures to assist researchers in managing this impact should be more a routine task. There is a growing consensus that research institutions should consider risks to researchers as a part of the risk assessment undertaken during ethics processes; this can be especially pronounced when these involve early career researchers for whom the institution has a duty of care ^(7)^. This is all the more important when many of the researchers are disaster-affected local residents and often targeted by the criticisms causing the above-mentioned survey fatigue, as was the case with the 3.11 disaster. Expanding the culture of disaster research to both facilitate understanding of how to work with the risk of vicarious trauma and how to connect with and contribute to communities can support researchers and the communities they work with ^(8), (9)^.

The third area for reflection―how researchers position the communities in their research― bridges the previous two points. In particular, the concept of vulnerability as applied to disaster-exposed peoples and places can be further interrogated. The formal research ethics process tends to locate vulnerability within the individual research participant. While this is undoubtedly important, other forms of vulnerability―particularly those faced at the community level―should also be a focus of ethical practice. This may also include, for instance, the ways in which disaster-impacted communities are framed within ongoing research dissemination. Emphasizing a status of “victim” can also exacerbate issues of stigmatization (for example in relation to discussions of nuclear radiation contamination in areas close to the Fukushima Daiichi power plant) ^(10)^. Such framings underestimate the resilience of people and communities in disaster recovery and re-emphasize hardships and suffering as primary narratives to describe the complex social realities of these communities. This effect can be further complicated by the politics of disaster reconstruction. In relation to 3.11, despite survey fatigue, survey evidence constructing the disaster-affected population as victims is perceived as being linked to the continued compensation ^(5)^.

It is critical to recognize the interwoven perspectives and roles of researchers and communities in disaster research and recovery. Comprehensively reflecting on the impact of the research community on disaster-affected populations will strengthen research practice as well as maximize positive outcomes for the communities themselves. We highlight these learnings from over a decade of disaster research at Fukushima and underscore their relevance to broader discussions of disaster research ethics.

## Article Information

### Conflicts of Interest

None

### Sources of Funding

This work was supported by [Great British Sasakawa Foundation Butterfield Award] grant number [B142].

### Acknowledgement

Many thanks to the participants of our project for their valuable time and reflections.

### Author Contributions

KH, SA, and AY conceptualized and produced the first draft of the paper; CL, ALL, and AO contributed to review and editing of the paper.

### Approval by Institutional Review Board (IRB)

This was approved by the University of Edinburgh School of Social & Political Science Research Ethics Committee (No. ID 284739).

### Disclaimer

Aya Goto is one of the Editors of JMA Journal and on the journal’s Editorial Staff. She was not involved in the editorial evaluation or decision to accept this article for publication at all.

## References

[1] Abeysinghe S, Leppold C. Ethical research practice in health and disasters. Int J Disaster Risk Reduc. 2023;92:103728.

[2] McCurry J. Fukushima water discharge approved. Lancet. 2023;402(10398):277-8. 37482063 10.1016/S0140-6736(23)01511-8

[3] Gaillard JC, Peek L. Disaster-zone research needs a code of conduct. Nature. 2019;575(7783):440-2. 31748720 10.1038/d41586-019-03534-z

[4] Yumiya Y, Goto A, Murakami M, et al. Communication between health professionals and community residents in Fukushima: a focus on the feedback loop. Health Commun. 2020;35(10):1274-82. 31167578 10.1080/10410236.2019.1625004

[5] Reich MR, Goto A. Towards long-term responses in Fukushima. Lancet. 2015;386(9992):498-500. 26251395 10.1016/S0140-6736(15)61030-3

[6] Smith E, Pooley JA, Holmes L, et al. Vicarious trauma: exploring the experiences of qualitative researchers who study traumatized populations. Disaster Med Public Health Prep. 2021;17:e69. 34955115 10.1017/dmp.2021.333

[7] Mukherji A, Ganapati NE, Rahill G. Expecting the unexpected: field research in post-disaster settings. Nat Hazards. 2014;73(2):805-28.

[8] Ness RB, Andrews EB, Gaudino JA Jr, et al. The future of epidemiology. Acad Med. 2009;84(11):1631-7. 19858828 10.1097/ACM.0b013e3181bbb4ed

[9] Murakami M, Tsubokura M. Deepening community-aligned science in response to wavering trust in science. Lancet. 2021;397(10278):969-70. 33714385 10.1016/S0140-6736(21)00358-5

[10] Tanaka M. The social amplification of stigma in the media after the Fukushima disaster. Routledge; 2022. Health, wellbeing and community recovery in Fukushima; p. 135-52.

